# Assessment of exopolysaccharide production by *Lactobacillus delbrueckii* subsp. *bulgaricus* ropy strain in different substrate media

**DOI:** 10.1002/fsn3.1452

**Published:** 2020-02-20

**Authors:** Ratmawati Malaka, Fatma Maruddin, Zaraswati Dwyana, Maynor V. Vargas

**Affiliations:** ^1^ Laboratory of Biotechnology of Milk Processing Department of Animal Science Faculty of Animal Science Hasanuddin University Makassar Indonesia; ^2^ Laboratory of Microbiology Department of Biology Faculty of Mathematic and Natural Sciences Hasanuddin University Makassar Indonesia; ^3^ Laboratory of Chemistry and Applied Biosciences National Technical University (UTN) Alajuela Costa Rica

**Keywords:** exopolysaccharide, *Lactobacillus delbrueckii* subsp. *bulgaricus*, reconstituted skim milk, ropy strain, soy milk whey

## Abstract

The aim of this research was to determine the optimal medium for Exopolysaccharides (EPS) production by a *Lactobacillus delbrueckii* subsp. *bulgaricus* ropy strain isolated from a locally produced commercial fermented milk, in reconstituted skim milk (RSM) 10% (w/v), milk whey (MW), and soy milk whey (SMW), under optimal growth conditions for this strain. Milk whey was made by coagulating fresh milk using papaya latex 3% (v/v); soy milk whey was obtained from tofu household industry. The chemical composition of the substrate media was determined by proximate analysis, and sterilization was accomplished in an autoclave at 121°C for 15 min. Culture media were inoculated with 1% (v/v) of a starter culture of *L. delbrueckii* subsp*. bulgaricus* and then incubated at 30°C for 16 hr. EPS production, lactic acid content, cell counting, and pH were determined after the media were cooled at 5°C. Findings showed that on the basis of the growth characteristics of *L. delbrueckii* subsp*. bulgaricus*, the best medium for EPS production was RSM 10% (258.60 ± 26.86 mg/L) compared to the milk whey (69.60 ± 9.48 mg/L) and soy milk whey (49.80 ± 9.04 mg/L).

## Graphical abstract

The aim of this research was to obtain the best medium for EPS production by *Lactobacillus bulgaricus* ropy strain in optimum growth conditions based on its growth characteristics in reconstituted skim milk RSM 10% (w/v), milk whey (MW), and soy milk whey (SMW). Result indicated that on the basis of the growth characteristics of *Lactobacillus delbrueckii* subsp. *bulgaricus*, the best medium for EPS production was RSM 10% (258.60 mg/L) compared to the milk whey (69.60 mg/L) and soy milk whey (49.80 mg/L).

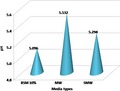

## 1 INTRODCUTION

*Lactobacillus bulgaricus* subsp. *bulgaricus* is used in the dairy industry to convert milk into yogurt (Ali et al., 2019; Bari et al., 2017; Chervaux, Ehrlich, & Maguin, 2000). Physiological processes during growth and reproduction of this kind of bacteria in a particular medium are not yet widely known or understood, especially in relation to its ability to produce Exopolysaccharides (EPS) as bacterial metabolites (Leroy & Vuyst, 2016; Petry, Furlan, Crepeau, Cerning, & Desmazeaud, 2000). These bacterial extracellular polymeric substances have currently become a subject of great interest among modern microbiologists and biotechnologists (Hererer, ElFallal, Abou‐Dobara, Toson, & Abdelaziz, 2018), especially EPS from lactic acid bacteria (LAB), such as *L. delbrueckii* subsp*. bulgaricus*, a starter culture of yoghurt (Bouzar, Cerning, & Desmazeaud, 1997). Extracellular polysaccharides (EPS) produced by certain microorganisms are currently being used in the food industry as stabilizers and binding agents, to increase viscosity and to improve the rheological properties of food products (Al‐Dhaheri et al., 2017; Bari et al., 2017; Cerning, Boullanne, Landon, & Desmazeaud, 1992; Galle & Arendt, 2014; Han et al., 2016; Malaka, 1997; Malaka, Maruddin, Baco, & Ohashi, 2019; Malaka, Ohashi, & Baco, 2013; Pachekrepapol, Lucey, Gong, Naran, & Azadi, 2017; Ruas‐Madiedo, Hugenholtz, & Zoon, 2002; Shi, Hou, Xu, Krogh, & Tenkanen, 2019; Trancoso‐Reyes, Gutiérrez‐Méndez, Sepúlveda, & Hernández‐Ochoa, 2014; Xu et al., 2019). EPS have also been investigated as antiviral (Katsuraya et al., 1995) and antitumor agents (Liu et al., 2018; Malaka, Abustam, & Baco, 2016), besides other beneficial health effects (Adebayo‐Tayo, Ishola, & Oyewunmi, 2018; Lobo, Gomes, Valdés, & Torino, 2019).

The composition of the culture media greatly affects the ability of microorganisms to metabolize specific nutrients in order to produce energy for their growth and reproduction needs. *Lactobacillus bulgaricus* subsp. *bulgaricus* ropy strain can produce polysaccharides making the yogurt thicker and finer in texture (Ali et al., 2019). For its optimal growth, *L. delbrueckii* subsp*. bulgaricus,* as a species of LAB, requires several types of nutrients. Some types of media such as milk or whey contain enough nutrients needed by LAB and are often used to produce important bacterial metabolites such as lactic acid, alcohols, acetic acid, and other value‐added substances of economic interest.

Considering that *L. delbrueckii* subsp*. bulgaricus* is normally present in fresh milk and that reconstituted milk (10% b/v RSM) resembles fresh milk, it was selected as a test medium. Some researchers use nonfat milk in order to maintain *L. delbrueckii* subsp*. bulgaricus*. On the other hand, milk whey (MW) is a waste that separates after curdling in cheese production and still contains enough nutrients to support microbial growth (Briczinski & Roberts, 2002). Furthermore, SMW is also a liquid waste that separates from the soy milk lumps during the manufacture of soybean tofu and contains nutrients needed for microbial growth. In addition, the nutritional composition of soy milk used in tofu production is almost the same as in mammalian milk.

For the optimization of this process, it is very important to select the medium that best stimulates and enhances the production of EPS by the *L. delbrueckii* subsp*. bulgaricus* ropy strain.

## 2 MATERIAL AND METHODS

### 2.1 Bacterial strains and fermentation conditions

*Lactobacillus delbrueckii* subsp. *bulgaricus* ropy strain was obtained from the collection of the Milk Processing Biotechnology Laboratory in the Faculty of Animal Husbandry in Hasanuddin University, Indonesia, which was isolated from a locally commercial ropy yoghurt and routinely propagated in 10% Reconstituted Skim Milk medium (RSM), twice a week (Malaka et al., 2016). The bacterial strain (0.1% inoculums w/v) was grown in sterile 10% (w/v) RSM (sterilized at 121°C, 15 min), and incubate data temperature of 37°C for 16 hr in order to enhance optimal cell growth.

Three batch fermentations without pH control were carried out in 100‐ml Erlenmeyer flasks containing 10% RSM, MW, and SMW media, respectively. The 10% (w/v) RSM media was prepared by dissolving 10 g of powdered skim milk in 90 ml of distilled water. Milk whey was obtained from a small‐scale Dangke cheese processing factory, and SMW was obtained from residual effluents of a small‐scale tofu processing factory. Chemical compositions of all three media were determined by proximate analysis. Media were sterilized at 121°C for 15 min, and after cooling to 30°C, they were inoculated with the starter culture of *L. delbrueckii* subsp*. bulgaricus* (0.1% w/v inoculum) and then incubated at 30°C for 16 hr in order to study EPS production. Samples were stored at 5°C before testing.

### 2.2 Growth measurement

Bacterial growth of *L. delbrueckii* subsp*. bulgaricus* was determined by measuring lactic acid production and pH decrease in the media. Lactic acid was determined by weighing 2.00 ± 0.05 g of culture media and titrating with 0.1 N NaOH using phenolphthalein as indicator.

The concentration of lactic acid was calculated using the formula (Marshall, 1993):

$$\%\;\text{Lactic Acid}=\frac{(\text{ml NaOH}\times \text{N NaOH})\times 0.09}{\text{Sample weight}\;(g)}\times 100\%$$

Counting of *L. delbrueckii* subsp*. bulgaricus* live cells was done by the dilution method using 0.9% NaCl solution and Bromocresol purple agar (BCPA) as growth medium. Serial dilutions of each sample were poured into petri dishes in duplicate (duplo) and then incubated at 37°C for 48 hr. Results were expressed as colony forming units (CFU/ml). pH measurements were carried out using a pH meter (Hanna pH meter Instrument 302 Portugal).

### 2.3 EPS production

EPS isolation was done from a cell‐free supernatant (centrifugation at 4,032 *g*). The supernatant was heated in a water bath at 65°C for 30 min (LTLT; low temperature long time pasteurization) to activate the enzyme able to degrade the polymer; afterward, papain enzyme was added as much as 1% to coagulate casein. Samples were then centrifuged at 6,000 rpm, and the supernatant was stored at 5°C for 24 hr. The polymer was precipitated with 95% cold ethanol in a 1:1 ratio (v/v), stored at 5°C for 24 hr, and then centrifuged at 7, 168 *g*. The precipitate was washed with deionized water three times to eliminate the remaining sugars from the culture medium, then freeze‐dried, and weighed as dried EPS powder. EPS production was expressed in mg/L units (modified from Cerning et al., 1992; Mozzi, Giori, Oliver, & Valdez, 1994).

### 2.4 Data analysis

Data obtained from the results of this study were processed by analysis of variance in a completely randomized design (CRD) with three treatments and five replications (Gaspersz, 1994; Hinkelmann & Kempthorne, 2007) using the SPSS program. Mathematical models were applied as follows:

$$Y_{\mathrm{ij}}=\mu +\alpha_{i}+\varepsilon_{\mathrm{ij}}$$

where *Y_ij_* = Value of observation in the experimental unit that receives treatment *i* (media type factor). *µ* = The average all of observations. *α* = Effect of media type on the percentage of lactic acid, pH, EPS and total *L. delbrueckii* subsp*. bulgaricus*. *ε_ij_* = Effect of random error on observation of the *j* which received treatment *i*.

When data analysis showed that there was a significant effect of the treatment, it was then evaluated by the smallest significant difference test (LSD). The significant difference was set at the 95% confidence level.

## 3 RESULTS AND DISCUSSION

### 3.1 Media composition

Table 1 shows that the protein content in both SMW and RSM 10% were very similar, but RSM 10% has a higher carbohydrate content in comparison with MW and SMW. In addition, mineral contents in the three culture media were almost the same and the substrates used for the production of EPS, lactic acid, and cell growth corresponded to the proteins, fats, carbohydrates, and minerals present in each media. Reconstituted skim milk medium (10%) mainly contains two types of proteins: serum proteins and casein. Casein consists of α‐, β‐, γ‐, and κ‐casein. Caseins are globular proteins, and all four types have a similar amino acid composition (Corredig, Nair, Li, Eshpari, & Zhao, 2019; Holt, Carver, Ecroyd, & Thorn, 2013). Whey protein contains β‐lactoglobulin and α‐lactalbumin, proteases (derivatives from β‐casein hydrolysis), and small amounts of protein derived from blood, serum albumin, and immunoglobulins (Mann, Athira, Sharma, Kumar, & Sarkar, 2019). Whey protein is a compact globular protein with a relatively uniform and denatured distribution at 60°C. Amino acids are essential for the growth of *L. delbrueckii* subsp*. bulgaricus*. Removal of several types of amino acids can inhibit the growth of *L. delbrueckii* subsp*. bulgaricus*; the effect is especially remarkable with asparagine, glutamine, and threonine, although aspartic acid, glutamic acid, or glycine only slightly affect the growth of these bacteria (Hebert, Raya, & Giori, 2004).

**Table 1:** Nutritional composition of three types of media used in EPS production by *Lactobacillus delbrueckii* subsp. *bulgaricus* ropy strain

Nutritional composition	Media type		
	Milk whey (MW)	Soy milk whey (SMW)	RSM 10%
Water (%)	96.19 ± 12.00	91.65 ± 13.30	90.40 ± 11.90
Energy value (Kcal)	63.85 ± 3.40	27.68 ± 3.90	35.80 ± 5.60
Protein (%)	1.97 ± 0.20	3.42 ± 0.90	3.53 ± 0.60
Fat (%)	0.62 ± 0.08	3.07 ± 0.50	0.10 ± 0.02
Carbohidrate (%)	0.43 ± 0.05	1.24 ± 0.08	5.19 ± 0.09
Mineral (%)	0.79 ± 0.07	0.62 ± 0.01	0.8 ± 0.01

Note

Analysis performed at the Laboratory of Animal Nutrition and Food of the Faculty of Animal Husbandry, Hasanuddin University.

The main carbohydrate present in milk corresponds to lactose with concentrations that vary between 4.2% and 5.0%. Lactose is a disaccharide containing α‐d‐glucose and β‐d‐galactose molecules (Varnam & Sutherland, 1994; Malaka & Abustam, 2004).

Major ionic species in milk include bicarbonate, chloride, citrate, calcium, magnesium, potassium, and sodium. All minerals are distributed between the soluble phase and the colloidal phase. The distribution of calcium, citrate, magnesium, and phosphate in the colloidal phase and soluble phase and their interaction with milk protein is very important for the stability of milk and milk products (Varnam & Sutherland, 1994). Minerals are needed in microbial metabolism as electron acceptors for the metabolism of glucose and other sugars.

Milk whey is a liquid residue obtained from cheese and casein production. Whey solids consist of 80%–90% protein and 10%–20% of soluble milk constituents such as lactose, vitamins, and minerals (Bylund, 1995). The lack of casein protein in milk whey arises from the fact that casein precipitates as curd, and the protein contained in whey corresponds to α‐lactalbumin and β‐lactoglobulin which are proteins needed to enhance the body's resistance to diseases.

On the other hand, SMW is a liquid residue that comes out as a by‐product of the curdling process of soy milk when making tofu (Li et al., 2013). Soymilk whey still contains nutrients such as crude proteins (4.62 ± 0.14%), total carbohydrates(8.77 ± 0.38%), ash content (5.14 ± 0.01%), and lipids (0.024 ± 0.001%) (Sobral, Ossa, Palazolo, & Wagner, 2018).

### 3.2 Effect of media type on microbial growth parameters

#### 3.2.1 Effect of media type on pH

**Figure 1:**
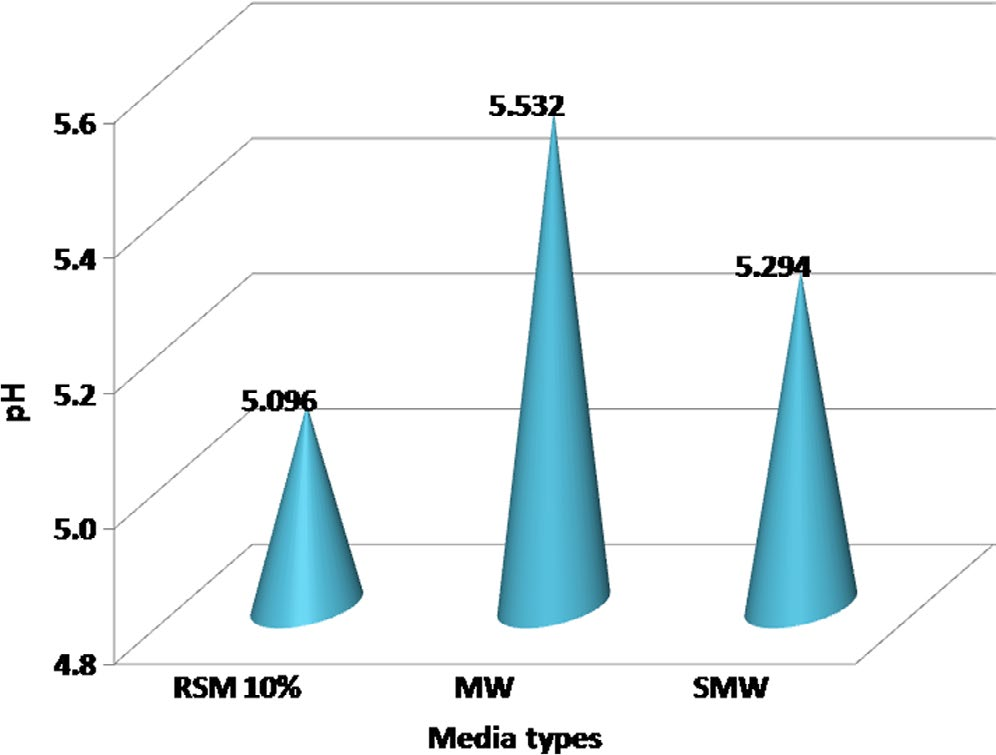
Effect of media type of *Lactobacillus delbrueckii* subsp. *bulgaricus* culture on pH of media. MW, milk whey; SMW, soy milk whey; RSM, reconstituted skim milk

The effect of media type on changes in pH values is shown in Figure 1. Among the three types of media, it can be seen that the 10% RSM media showed the fastest decrease in pH (lowest pH value) which corresponded to an average value of 5.096, while the lowest pH decrease (corresponding to the final highest pH value) at MW media was 5.532; pH in SMW media reached to 5.294. The slower decrease in pH in whey is presumably caused by the lower content of carbohydrates (Table 1), so that just a little amount of glucose is converted into lactic acid. In glucose metabolism through the glycolysis pathway after passing through the pyruvate pathway, the enzyme lactate dehydrogenase will transform pyruvic acid into lactic acid (Vicente et al., 2018) and that make the pH of the media decrease.

Analysis of variance showed that the type of media had a very significant effect (*p* < .01) on pH decrease. Furthermore, the least significant difference (LSD) testing was performed showing a very significant difference (*p* < .01) between the three types of media on the final pH of the media, and the pH decrease observed. Under conditions of high carbohydrate contents, especially at 10% RSM, pyruvic acid acts as a hydrogen recipient to produce lactic acid through its reduction by NADH_2_. Transformation of glucose into pyruvic acid release H^+^ ions needed in the process of changing pyruvic acid to lactic acid. The presence of carbohydrates in the 10% RSM media as high as 5.19% causes significative differences in the final acidity of 10% RSM media, in comparison with MW and SMW media.

#### 3.2.2 Effect of media type on lactic acid production

**Figure 2:**
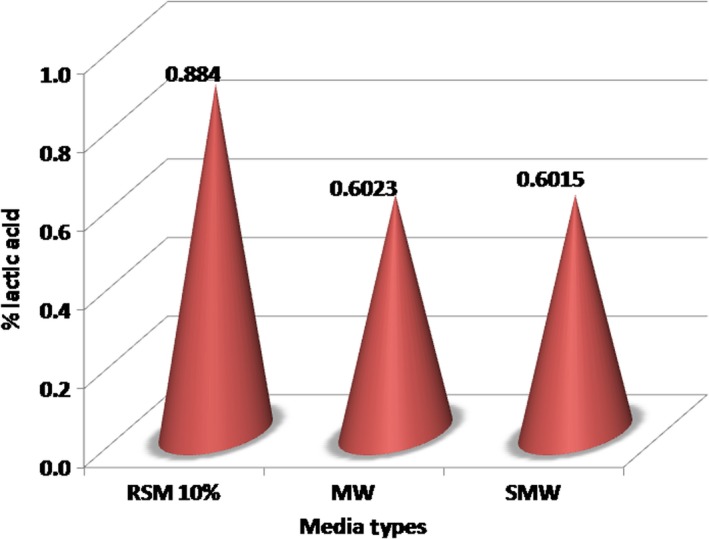
Effect of media type of *Lactobacillus delbrueckii* subsp. *bulgaricus* culture on % lactic acid. MW, milk whey; SMW, soy milk whey; RSM, reconstituted skim milk

Figure 2 shows the relationship among the three types of media as regards of lactic acid production. The highest production rate was found in 10% RSM, which ranged from 0.824% to 0.930%, with an average final concentration of 0.884%. As for MW and SMW, lactic acid production was very close, reaching an average of 0.602%. Lactic acid production in MW and SMW media was lower because a reduced carbohydrate content in comparison with the 10% RSM medium.

During lactic acid fermentation, lactose undergoes a hydrolysis step to produce monosaccharides in the glycolysis pathway (Berg, Tymoczko, & Stryer, 2002). In some cases, disaccharides breakdown through phosphohydrolase, and one sugar molecule turns into a free monosaccharide and the other one becomes a monosaccharide phosphate. *Lactobacillus bulgaricus* as one type of BAL generally produces a system of lactose phosphotransferase where lactose will enter the cytoplasm as lactose phosphate which will join phospho‐β‐d‐galactosidase to produce glucose and galactose‐6‐phosphate. Glucose phosphorylase is metabolized by glucokinase via the glycolytic pathway. Galactose‐6‐phosphate is metabolized via the tagatose‐6‐phosphate pathway (Neves, Pool, Kok, Kuipers, & Santos, 2005). The low production of lactic acid in whey is due to the very limited amount of lactose in MW. Likewise, lactic acid in SMW is produced from a type of carbohydrate different to that of lactose type.

In this study, substrates used for EPS production by *L. delbrueckii* subsp*. bulgaricus* were proteins, carbohydrates, and minerals, as shown in Table 1. Milk whey media has a carbohydrate content of 0.43 g substrate/100 ml that can be used as a substrate to produce lactic acid. Therefore, *L. delbrueckii* subsp*. bulgaricus* is theoretically able to convert 1 g of substrate to 0.602/0.43, that is 1.4% lactic acid or 0.014 g (1.4 mg) lactic acid on 100 ml basis of MW media. The SMW media contains 1.24 g carbohydrate/100 ml, which can be transformed by *L. delbrueckii* subsp*. bulgaricus* to 0.602/1.24, that is 0.49% or 0.49 mg lactic acid. Reconstituted skim milk contains carbohydrate substrate as much as 5.19%. This concentration implies that *L. delbrueckii* subsp*. bulgaricus* can change 1 g of substrate to 0.884/5.19, that is 0.17% lactic acid or 0.0017 g (0.17 mg) lactic acid in 100‐ml milk media.

Based on analysis of variance, it was corroborated that the media type has a very significant effect (*p* < .01) on lactic acid production (%). The LSD test showed that lactic acid from 10% RSM medium was significantly different from lactic acid from both MW and SMW, but between MW and SMW there was no significative difference. From several studies (Ramos, Boels, Vos, & Santos, 2001), it is known that the synthesis of EPS in nutritive media shows that there is a close relationship between cell growth, lactic acid production, and EPS production, which is associated with the presence of certain nutrients, especially carbohydrates as the main medium and protein as a catalyst and minerals as cofactors. In media with low content of carbohydrates, *Lactobacillus bulgaricus* generates a limited amount of lactic acid (Percuma, Hebert, Mozzi, & Valdez, 2008).

#### 3.2.3 Effect of media types on the growth of *L. delbrueckii* subsp. *bulgaricus*

**Figure 3:**
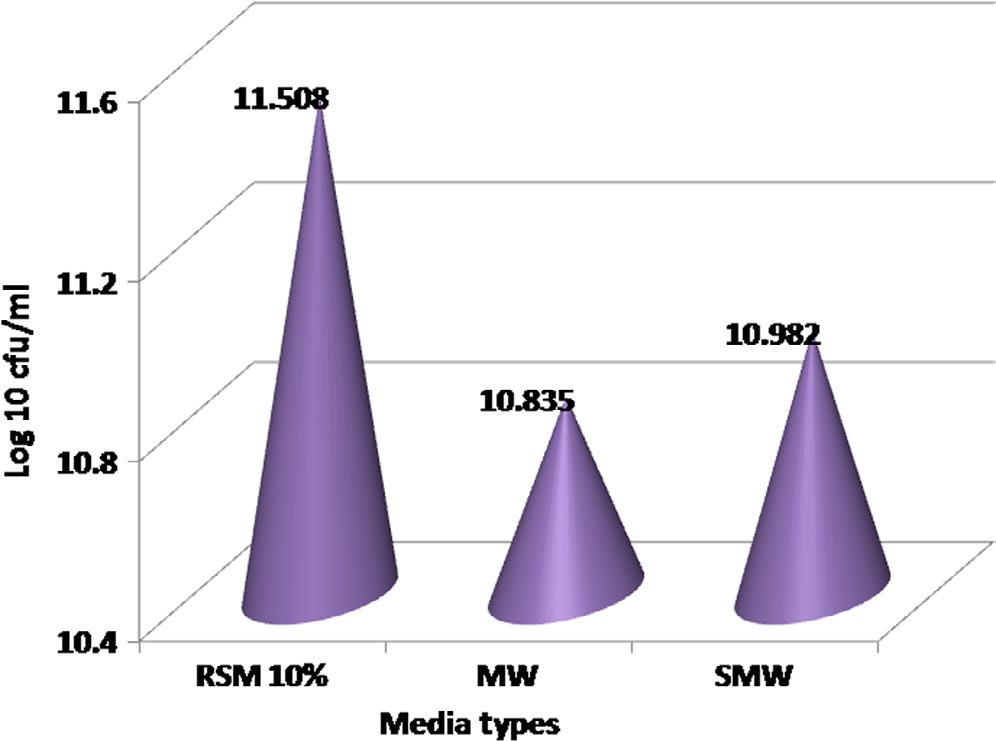
Effect of media type of *Lactobacillus delbrueckii* subsp. *bulgaricus* culture on bacterial number. MW, milk whey; SMW, soy milk whey; RSM, reconstituted skim milk

The effect of the type of media on bacterial growth, determined by the number of live bacteria, is shown in Figure 3. Maximum growth after incubation at 30°C for 16 hr reached an average of 11.508 (log CFU) in 10% RSM media. In whey media, the amount of *L. delbrueckii* subsp*. bulgaricus* reached 10.835 (log CFU/ml) and in the SMW medium it was 10.982 (log CFU/ml). These numbers indicate that these bacteria have reached the stationary growth phase.

Microbial growth begins with the decomposition of the substrate constituted by proteins, minerals, and carbohydrates. The oxaloacetate decarboxylase that produces pyruvate will join through the diacetyl/acetoin pathway*. Lactobacillus bulgaricus* as homofermentative LAB, on the pyruvate pathway, will produce lactic acid and more ATP is formed; thus, the glucose utilization becomes more efficient and stimulates the growth rate (Papagianni, 2012). This can be seen in the 10% RSM medium, which presents the most efficient growth. Growth depends on the ability of the cell to form new protoplasm from nutrients found in the growth substrate. It begins with an increase in cell mass and the number of ribosomes, duplication of ribosomes, synthesis of new cell walls and plasma membranes, separation of two chromosomes, septal formation, and cell division (Nair, 2010). Carbon derived from the carbohydrate substrate in the media is needed by bacteria as an energy source. Amino acids are used as growth factors for the formation of nucleic acids and minerals. They are needed as electron acceptors and as coenzymes in cell metabolism (de Vladar, 2012).

Based on the variance analysis, the type of media accounted for a very significant effect (*p* < .01) on the total number of live bacteria of *L. delbrueckii* subsp*. bulgaricus*. The Smallest Significant Difference Test (SSD) indicated that the bacterial counts in the three types of media were significantly different, although closer between 10% RSM and SMW. Under conditions of insufficient availability of carbohydrates as a carbon source for cell growth and metabolism, these bacteria can use other carbon sources present in the media, such as proteins, acetic acid, or even carbonic acid. Although the bacteria counts were large, the decrease in pH and the production of lactic acid is rather dependent on the presence of lactose or other carbohydrates contained in the media. Therefore, even though the number of bacteria was rather large, pH value does not necessarily decrease to 4.5 or lactic acid concentration increase to 0.8%–1.6%, although *L. delbrueckii* subsp*. bulgaricus* reaches this pH value and lactic acid concentration in the milk medium.

EPS production in 10% RSM with a total substrate content of 9.5 g/100 ml can produce as many as 11.50^8^ cells (log CFU/ml). The initial cell number in the inoculum was 10^5^ CFU/ml or 10^7^/100 ml, so the increase in bacterial population was 3.2 × 10^13^–10^7^ = 3.2 × 10^13^. As a result, bacterial cell number reached as much as 3.2 × 10^13^/9.5, that is 3.37 × 10^11^/g substrate. In MW media with substrate concentration of 3.187 g/100 ml and cells count of 10.835 log CFU/ml or 6.84 × 10^10^ (6.84 × 10^12^ CFU/100 ml), it was determined that cell production reaches as much as 6.84 × 10^12^/3.187, that is 2.1 × 10^12^ CFU/g substrate. Finally, in SMW media with a substrate concentration of 5.284 g/100 ml its bacterial counts reach as much as 10.982 log CFU/ml, that is 9.6 × 10^10^ (9.6 × 10^12^ CFU/100 ml). Thus, in SMW media, 1 g of substrate can produce 9.6 × 10^12^/5.284, that is 1.82 × 10^12^ CFU/g substrate.

Based on substrate conversion, the best medium where substrate is converted into cells was observed in whey medium.

#### 3.2.4 Effect of media type on EPS production

**Figure 4:**
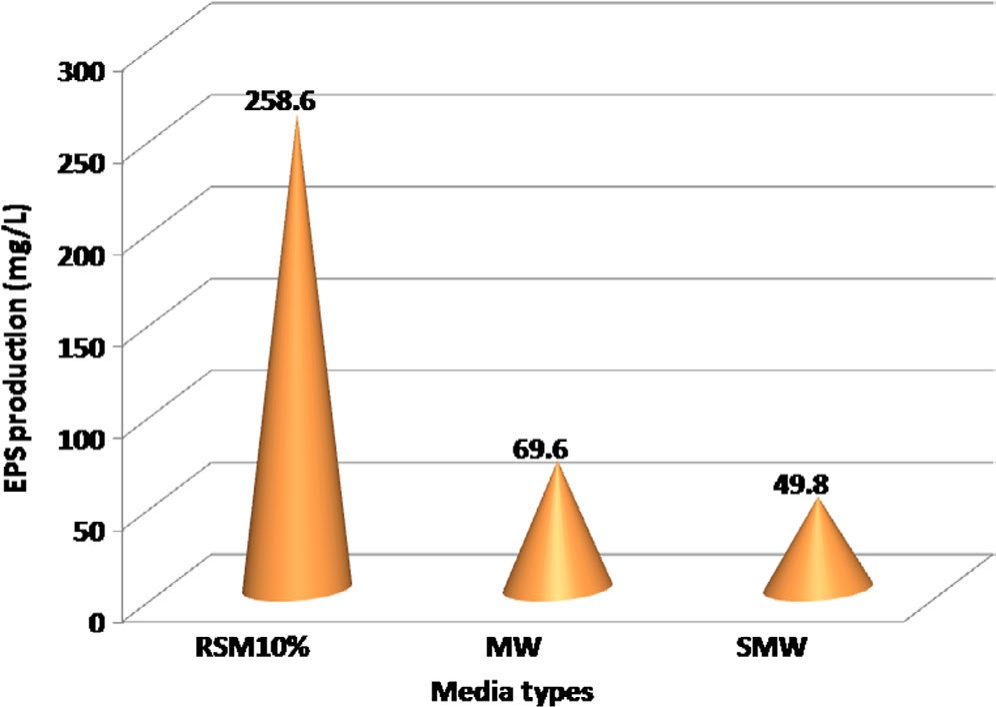
Effect of media type of *Lactobacillus delbrueckii* subsp. *bulgaricus* culture on EPS production. MW, milk whey; SMW, soy milk whey; RSM, reconstituted skim milk

Figure 4 shows that the maximum EPS production took place in the 10% RSM medium, with an average of 258.6 mg/L (ranging between 231 and 293 mg/L), while it only reached an average of 69.6 mg/L in the WM medium and an average of 49.8 mg/L in the SMW medium. This shows that in relation to EPS production, appropriate conditions are required to support the synthesis besides the need of a main carbon source for polymerization during the synthesis of polysaccharides.

Variable fingerprint analysis shows that the type of media had a very significant effect (*p* < .01) on EPS production. The LSD test showed that the 10% RSM media was significantly very different (*p* < .01) in comparison with the MW medium and the SMW medium. This was probably because the carbon content in the RSM medium was 10% higher in comparison with other media. Similarly, Briczinski and Roberts (2002) demonstrated that *L. delbrueckii* subsp*. bulgaricus* was able to form between 95 and 110 mg EPS/L in hydrolyzed whey. However, it turns out that MW and SMW media were not significantly different for EPS production. *Lactobacillus delbrueckii* subsp*. bulgaricus* can metabolize glucose from the decomposition of milk lactose, so that it can use this carbon source for the EPS production (Chervaux et al. (2000).

In substrate conversion of whey media (Table 2) based on protein, carbohydrates, and minerals contents, a concentration of 3.187 g/100 ml produces as much as 69.6 mg EPS/L (equivalent to 6.96 mg/100 ml or 0.00696 g/100 ml). Thus for each gram of substrate, it can be produced as much as 0.0696 g EPS/3.187 g substrate, that is 2.18 mg/g substrate. In SMW medium with a total substrate of 5.287 g/100 ml, it can be produced as much as 49.8 mg/L (equivalent to 4.98 mg/100 ml or 0.00498 g/100 ml). Thus, in the SMW medium, EPS production reaches as much as 0.0498/5.287, that is 9.42 mg/g substrate. While in 10% RSM media with a substrate concentration of 9.5 g/100 ml, EPS can be produced as much as 258.6 mg/L (equivalent to 25.86 mg/100 ml or 0.02586 g/100 ml). Thus in 10% RSM it can be produced as much as 0.2586 g EPS/9.5 g substrate, that is 27.2 mg EPS/g substrate.

**Table 2:** Value of 1 g conversion of substrate on 10% SSR media, whey, and tofu water into lactic acid, cells, and EPS

Media types	Substrates (%)	Lactic acid (mg)	Log CFU	EPS (mg/g)
SSR 10%	9.5	0.17	3.37 × 10^11^	2.70^a^
Whey	3.187	1.40	2.1 × 10^12^	2.18^b^
Tofu whey	5.284	0.49	1.82 × 10^12^	0.94^c^

Note

Different letters in the same column show significant differences.

Exopolysaccharide (EPS) production occurs during different growth phases under conditions that vary depending on the type of microorganism. The heteropolysaccharide synthesis is different from that of monosaccharides (Ates, 2015). The synthesis of EPS in nutrient media shows that the polymer is continuously excreted shortly after growth and during the cell division. The specific enzymes that play a role in the biosynthesis of EPS are the glycosyltransferase. This enzyme is needed to assemble repetitive units used in long chain determination, during the polymerization of nucleotide sugars as unique monomers precursors for EPS. The gene for EPS production by *L. bulgaricus* is produced as a doubling reaction through chromosomes (Zeidan et al., 2017).

Exopolysaccharides, in the form of heteropolysaccharides, are synthesized with precursor polymerases formed in cytoplasmic cells. Nucleotide sugars play an important role in carrying isoprenoid lipids located in the cytoplasm. The synthesized cell components compete with EPS formation during different growth phases (Madhuri & Prabhakar, 2014), what can be noticed by the occurrence of lactic acid production which causes a decrease in pH, the occurrence of cell growth and EPS production during the incubation process of the growth media. This explains why the conversion of the substrate from each media is different according to the different chemical compositions.

Conversion value of 1 g of substrate into lactic acid products, cell counts, and EPS production are summarized in Table 2. It was observed that the values for the substrate conversion to EPS are best accomplished in a 10% RSM media.

Lactose, as the main carbohydrate in milk, enters *L. bulgaricus* cells through active transport through the lactose‐phosphoenolpyruvate (Lac‐PEP) enzyme, and glucose and galactose‐6P are broken down into glucose and galactose. Afterward, glucose enters the UDP‐glucose pathway for EPS formation. Galactose‐1‐phosphate then enters the Leloir pathway into tagatose‐5P with an isomerase reaction and then enters the Embden‐Meyerhof pathway or pyruvate pathway by producing lactic acid and the formation of ATP for cell reproduction (Ramos et al., 2001). Deficiency of nitrogen, phosphorus or sulfur sources in media containing carbohydrates increases EPS production. Glucose‐6P is a key metabolite at the branching point between EPS glycolysis and biosynthesis. If metabolism follows the glycolysis pathway, glucose metabolism will lead to the formation of lactic acid which causes a decrease in pH (Boels, Kleerebezem, & Vos, 2003).

Rimada and Abraham (2003) found that kefir granules in whey deproteinization media had higher EPS production compared to milk media which in the whey media was 247 mg/L and in milk media was 218 mg/L. This is due to the differences in the type of starter used. *Lactobacillus delbrueckii* subsp*. bulgaricus* was used in this study, while Rimada and Abraham used kefir granules consisting of various types of LAB and yeast. The type of carbohydrate in the form of monosaccharide or disaccharide plays an important role in the synthesis of EPS, as well as during the formation of polymers containing other glycans. The role of nucleotide sugars is activated by monosaccharides and cannot be transferred directly to acceptors during polymer synthesis, but it can be activated by free phosphorus and minerals that require energy and enzymes. Energy and enzymes are formed during cell metabolism by using substrates in the media (Bar‐Peled & O'Neill, 2011).

## 4 CONCLUSION

The best growth medium used for the production of EPS by *Lactobacillus delbruecki* subsp*. bulgaricus* is 10% RSM (258.60 ± 26.86 mg/L) compared to MW (69.60 ± 9.48 mg/L) and SMW (49.80 ± 9.04 mg/L). This result is supported by its growth characteristics which show that in RSM medium the microbial activity is maximal (cell count 11.5030 ± 0.0073 log CFU/ml, lactic acid 0.884 ± 0.004%) compared to MW media (cell count 10.833 ± 0.005 log CFU/ml, lactic acid 0.602 ± 0.004%) and SMW (11.19 ± 0.4011 log CFU/ml, lactic acid 0.602 ± 0.008%). Milk whey can be used for EPS production with enrichment of culture media, and SMW can be used as a medium for microbial and LAB cell production.

## CONFLICT OF INTEREST

The authors declare that they do not have any conflict of interest.

## ETHICAL APPROVAL

This study does not involve any human or animal testing.

## INFORMED CONSENT

This study does not require informed consent because it does not use humans as research subjects.
